# Pragmatic Implementation of a Comprehensive Dementia Care Management Program in a Large Health System: The Cedars‐Sinai C.A.R.E.S. Program Preliminary Data

**DOI:** 10.1002/alz.089549

**Published:** 2025-01-09

**Authors:** Zaldy S Tan, Nabeel Qureshi, Erica Spivack, Deana Rhinehart, Dyane Gatmaitan, Augustine Guinto, Sarah Kremen, Nancy Sicotte

**Affiliations:** ^1^ Cedars‐Sinai Medical Center, Los Angeles, CA USA

## Abstract

**Background:**

The United States faces a growing challenge with over 6.5 million people living with dementia (PLwD). PLwD and their caregivers struggle with cognitive, functional, behavioral, and psychosocial issues. As dementia care shifts to home settings, caregivers receive inadequate support but bear increasing responsibilities, leading to higher healthcare costs. In response, the Centers for Medicare & Medicaid Services (CMS) introduced the Guiding an Improving Dementia Experience (GUIDE) Model. The study explores the real‐world implementation of the Cedars‐Sinai C.A.R.E.S. Program, a pragmatic dementia care model, detailing its recruitment process and initial outcomes.

**Methods:**

The Cedars‐Sinai C.A.R.E.S. Program was integrated into the Epic electronic health record system, and focused on proactive patient identification, engagement, interdisciplinary collaboration, care transitions, and ongoing care management. Eligible patients aged 65 and older with a dementia diagnosis were identified through electronic health record and invited to join the program. Nurse practitioners with specialized training in dementia care performed comprehensive assessments using the novel CEDARS‐6 tool, leading to personalized care plans developed in consultation with primary care providers. Patients benefit from a multidisciplinary team and support from care navigators.

**Results:**

Of the 781 eligible patients identified, 431 were enrolled in the C.A.R.E.S. Program. Enrollees were racially diverse, with lower caregiver strain and patient behavioral and psychological symptoms of dementia (BPSD) severity compared to other programs. Healthcare utilization, including hospitalizations, emergency department (ED) admissions, and urgent care visits showed a downward trend over time. Completion of advanced directives and Physician Order of Life Sustaining Treatment (POLST) increased after enrollment.

**Conclusion:**

The Cedars‐Sinai C.A.R.E.S. Program offers a promising approach to dementia care. Its real‐world implementation demonstrates the feasibility of enrolling a diverse population and achieving positive outcomes for PLwD and their caregivers, supporting the goals of national dementia care initiatives.

